# Evidence or no evidence for essential fatty acids in the treatment of autism spectrum disorders?

**DOI:** 10.3389/fnut.2023.1251083

**Published:** 2023-08-31

**Authors:** Rouzha Zlatanova Pancheva, Silviya Nikolova, Asena Serbezova, Krassimira Zaykova, Desislava Zhelyazkova, Lubomir Dimitrov

**Affiliations:** ^1^Department of Hygiene and Epidemiology, Faculty of Public Health, Medical University of Varna “Prof. Dr. Paraskev Stoyanov”, Varna, Bulgaria; ^2^Department of Social Medicine, Faculty of Public Health, Medical University of Varna “Prof. Dr. Paraskev Stoyanov”, Varna, Bulgaria; ^3^Department of Health Policy and Management, Faculty of Public Health, Medical University of Sofia, Sofia, Bulgaria; ^4^Faculty of Medicine, Medical University of Varna “Prof. Dr. Paraskev Stoyanov”, Varna, Bulgaria

**Keywords:** essential fatty acids, autism, supplementation, child, intervention

## Abstract

Autism spectrum disorders (ASDs) are a group of neurodevelopmental disorders that affect social communication, behavior, and sensory processing, in which PUFAs are considered important. This mini-review article aims to investigate the current evidence regarding the use of essential fatty acids (EFAs) in the treatment of autism spectrum disorders (ASDs). The study examines various research studies, related to EFAs, their benefits, and their role in ASD treatment. The article focuses on exploring the potential mechanisms underlying the effects of EFAs on ASDs, including their anti-inflammatory, antioxidant, and neuroprotective properties. Furthermore, the study discusses limitations and challenges associated with the use of EFAs in ASD treatment, including variability in dosage and duration of treatment. The results of this review indicate that while some studies suggest a positive effect of EFAs on ASD symptoms, there is currently insufficient evidence to support their routine use as a stand-alone treatment for ASD. The need for further research to better understand the potential benefits and limitations of EFAs in ASD treatment is highlighted.

## Introduction

Autism spectrum disorders (ASDs) are a group of neurodevelopmental disorders that affect social communication, behavior, and sensory processing. The prevalence of ASDs has increased significantly in recent years, with current estimates suggesting that 1 in every 36 (2.8%) 8-year-old children was estimated to have ASDs in 2020 (1).

Despite the growing prevalence of ASDs, effective treatments for the disorder are limited. Traditional therapies, such as behavioral interventions and medications, have shown some efficacy, but they often have significant limitations and may not be suitable for all individuals with ASDs (2). Therefore, there is a need for novel treatment approaches that can effectively address the core symptoms of ASDs.

One potential avenue for exploring new treatments for ASDs is through the use of dietary supplements (3). In particular, there has been growing interest in the potential role of fatty acids in the treatment of ASDs (1). Fatty acids are essential components of cell membranes and play important roles in the development and function of the brain (1).

Research in recent years has suggested that certain types of fatty acids, such as omega-3 and omega-6 fatty acids, may have therapeutic potential for individuals with ASDs (4). A high omega-6/omega-3 ratio in the cell membrane has been associated with inadequate brain development (5). This ratio [known as the “fatty acid (FA)” index] has started to be used as a biomarker of treatment efficacy in human diseases. The evidence supporting the use of fatty acids in the treatment of ASDs is still evolving, and there is a need for a comprehensive review of the available literature (6).

Therefore, this research article aimed to analyze the evidence supporting the use of fatty acids in the treatment of ASDs. This mini-review will examine the current literature on fatty acids and ASDs, with a focus on the potential benefits and limitations of fatty acid supplementation as a treatment avenue for children with autism.

## Essential fatty acids and brain development

Polyunsaturated fatty acids (PUFAs) are significant components of phospholipids, which are required for cell membrane structure and function. Linoleic acid (LA), an omega-6 acid, α-linolenic acid (ALA), an omega-3 acid, and their metabolic derivatives, namely arachidonic acid (AA), eicosapentaenoic acid (EPA), and docosahexaenoic acid (DHA), are essential structural and functional components of cellular and intracellular membranes in the human body (7).

Docosahexaenoic acid (DHA) is found in high concentrations in neural tissue, and research indicates that omega-3 fatty acids are crucial for the growth and functional development of the brain. Additionally, omega-3 fatty acids have anti-inflammatory effects (8).

The influence of essential fatty acids (EFAs) on brain development has been a subject of significant research interest. EFAs, including omega-3 and omega-6 polyunsaturated fatty acids (PUFAs), play key roles in various biological processes, including neuronal growth, synaptogenesis, and neurotransmitter signaling (9). Understanding the interactions between EFAs and brain development is crucial for gaining insights into their potential implications for neurodevelopmental disorders, such as autism spectrum disorders (ASDs).

During critical periods of brain development, EFAs serve as structural components of cell membranes and contribute to the formation and function of neural networks (10). Omega-3 PUFAs, such as docosahexaenoic acid (DHA), are particularly abundant in the brain and are crucial for neuronal membrane integrity, synaptic plasticity, and neurogenesis (11). Omega-6 PUFAs, such as arachidonic acid (AA), also play vital roles in brain development, modulating inflammatory responses and gene expression related to neuronal development (11).

Research investigating the impact of EFAs on brain development, including animal studies and observational studies in humans, has provided valuable insights. For instance, studies have shown that EFA deficiencies during pregnancy and early postnatal life can negatively affect brain development and cognitive function (12). Additionally, prenatal exposure to appropriate levels of omega-3 PUFAs has been associated with improved cognitive and behavioral outcomes in an offspring (13).

The interplay between EFAs and brain development in the context of ASDs has also been explored. Evidence suggests that individuals with ASDs may have altered EFA metabolism and imbalances in the omega-6 to omega-3 ratio (8). These imbalances may contribute to disruptions in neuronal signaling, synaptic plasticity, and inflammation, potentially influencing ASD symptomatology.

Furthermore, DHA is involved in cognitive functions, neurite formation, membrane fluidity, neurotransmission, endothelial function, neuronal survival, and neurodegeneration prevention. One study by Parletta et al. (14) reported lower levels of omega-3 PUFA EPA and DHA and a higher AA/EPA ratio in children with ADHD and ASD compared with typically developing controls, and these levels were associated with greater severity of symptoms. Individuals with ASD have altered PUFA metabolism, which results in increased production of proinflammatory cytokines, increased oxidative stress, and an imbalance in the formation and action of neurotransmitters (7).

While the relationship between EFAs and brain development shows promising scientific advances, it is important to note that the available evidence is not yet conclusive. Further research, including randomized controlled trials and longitudinal studies, is necessary to better understand the specific mechanisms by which EFAs interact with brain development and their potential implications for ASDs.

## Methodology

The methodology for this narrative review adhered to the Scale for the Quality Assessment of Narrative Review Articles (SANRA) guidelines (15). A comprehensive literature search was conducted in May–June 2023 across several electronic databases, including PubMed, Web of Science, and Scopus. The search strategy used the following keywords and boolean operators: “autism” OR “ASD” AND “omega-3′” OR “omega-6′” AND “supplementation” OR “effect” OR “intake”.

The initial search yielded a total of 639 articles. The articles were screened for duplicates, resulting in the exclusion of several duplicate entries. The subsequent screening was based on the titles and abstracts of the remaining articles, with the exclusion of reviews and non-relevant articles such as studies with no comparison or control group or qualitative-only designs. The inclusion criteria for the review were as follows: (1) randomized controlled trials (RCTs) or pre-post studies involving children with autism or ASD; (2) study participants in the study samples had to be under 18 years of age; (3) studies were included if they were reported on EFA outcomes; (4) if studies were interventions; and (5) articles had to be published in English.

The exclusion criteria were as follows: (1) studies focusing exclusively on animal models; (2) studies with no pre-post design and comparison groups; (3) review articles; and (4) articles in other than the English language.

Following this rigorous screening process, a total of 12 studies met all the inclusion criteria and were included in the final review. The selected studies underwent a thorough analysis and synthesis of their findings to inform the conclusions of this narrative review.

## Supplementation with omega-3 and omega-6 fatty acids in ASD

Table 1 provides a summary of studies and a comprehensive overview investigating the effects of omega-3 and omega-6 fatty acid supplementation on autism spectrum disorder (ASD) symptoms and behaviors.

**Table 1:** Summary of studies included in the review.

**References**	**Country**	**Sample size**	**Design of the study**	**Variables/outcome measures**	**Dosage**	**Duration**	**Improvement in ASD symptoms**	**Statistical significance**
Amminger et al. (16)	Australia	13	Double-blind, randomized, placebo-controlled trial	ASD symptoms, psychopathology	1.5 grams/day of omega-3	6 weeks	Improved hyperactivity and stereotypy	Yes
Bent et al. (17)	USA	27	Double-blind, randomized, placebo-controlled trial	Hyperactivity, stereotype	1.3 grams/day of omega-3	6 weeks	Improvement in hyperactivity, as measured by the Aberrant Behavior Checklist	Yes
Bent et al. (18)	USA	57	Randomized clinical trial		1.3 g of omega-3 fatty acids or an identical placebo	6 weeks	No statistically significant reduction in hyperactivity	No
Boone et al. (19)	Ireland	31	Double-blind randomized clinical trial	Caregiver-reported behavior and sleep in toddlers born at ≤ 29 weeks gestation	706 mg total omega-3 fatty acids: 338 mg EPA, 225 mg DHA; 280 mg total omega-6 fatty acids: 83 mg GLA; and 306 mg total omega-9 fatty acids	12 weeks	Mixed effects on measures of caregiver-reported behavior and sleep	Yes/no
de la Torre-Aguilar et al. (20)	Spain	54	Double-blind, randomized placebo-controlled	Plasma lipids, cytokines, and FA profiles in plasma and erythrocytes at baseline and after 6 months of treatment in ASD children and at baseline in the reference group.	252 mg DHA, 60 mg EPA	6 months	No clinical improvement	No
Doaei et al. (21)	Poland	54	Double-blind randomized clinical trial	Social, verbal, and behavioral activities in ASD children	1,000 mg/day of omega-3	8 weeks	Improved stereotype behaviors, social communication	Yes
Sheppard et al. (22)	USA	31 preterm born	Double-blind randomized controlled trial	Language abilities in children at risk for ASD	338 mg EPA, 225 mg DHA, 280 mg total omega-6 fatty acids (including 83 mg GLA), and 306 mg total omega-9 fatty acids/day	3 months	Increase in gesture use for children in the treatment group	Yes
Mazahery et al. (23)	New Zealand	73	Double-blind, randomized, placebo-controlled trial	Vitamin D and omega-3 supplementation; ASD symptoms (ATEC scores)	610 mg EPA, 405 mg DHA/day	16 weeks	Improvement in some core symptoms of ASD but no definitive conclusions	Yes/No
Parellada et al. (5)	Spain	68	Randomized crossover, placebo-controlled study	Erythrocyte membrane FA composition, plasma antioxidant status (TAS), social responsiveness and clinical global impression severity	962 and 1,155 mg/d for children and adolescents, respectively	8 weeks	Improved erythrocyte membrane without changing TAS; significant improvement in social motivation and social communication subscale scores.	Yes
Keim et al. (24)	USA	31	Double-blind randomized controlled trial	ASD symptoms and related behaviors, as reported by parents	338 mg EPA, 225 mg DHA, and 83 mg GLA/day	90 days	ASD symptoms reduced in the group receiving Omega-3-6-9 junior	Yes
Voigt et al. (25)	USA	48	Double-blind, randomized, placebo-controlled trial	Autism symptoms, maladaptive behaviors	200 mg/day of DHA	12 weeks	Teachers reported a higher average rating of functional communication; opposite for parent reported social skills	Yes/no
Yui et al. (26)	Japan	13	Double-blind, placebo-controlled, randomized trial	Social interaction, communication	40 mg of DHA, 40 mg of ARA/day	16 weeks	Improved social withdrawal and communication	Yes

In total, 12 clinical trials across seven countries were considered, with participant sample sizes ranging from 13 to 73. In terms of study design, all trials followed a double-blind, randomized, placebo-controlled trial design, with the exception of one (18) that was simply described as a randomized clinical trial. One study was a crossover trial (5). The duration of the selected studies ranged from 6 weeks to 6 months, with dosages varying considerably between the trials.

Significant ASD symptom improvements were reported in seven out of 12 studies. For instance, Amminger et al. (16) in Australia noted improvement in ASD symptoms and psychopathology after administering 1.5 g/day of omega-3 over a 6-week period. Similarly, Boone et al. (19) in Ireland reported positive behavioral and sleep changes in toddlers born at ≤ 29 weeks' gestation following 12 weeks of omega-3 supplementation. Furthermore, in Poland, Doaei et al. (21) demonstrated improved social, verbal, and behavioral activities in ASD children after providing them with 1,000 mg/day of omega-3 for 8 weeks. Similarly, in the United States, Sheppard et al. (22) observed reduced ASD symptoms and increased gesture use, respectively, in their study participants after supplementing them with a combination of omega-3, omega-6, and omega-9 fatty acids. Parellada et al. (5) reported significant improvement in social motivation and social communication subscale scores, with a moderate-to-large effect size (*p* = 0.004, *d* = 0.73 and *p* = 0.025, *d* = 0.79, respectively).

On the contrary, in Spain, two studies conducted by Bent et al. (18) and de la Torre-Aguilar et al. (20) did not find significant behavioral improvements despite the supplementation with omega fatty acids. Two studies conducted by Mazahery et al. (23) and Voigt et al. (25) in the USA and one study by Yui et al. (26) in Japan showed mixed or questionable effects. The longest supplementation study was performed on Spanish children with ASD, who exhibited an appropriate omega-3 FA status in plasma and erythrocytes without clinical improvement of ASD, or a better anti-inflammatory or fatty acid state has been found after an intervention with DHA/EPA for 6 months (20).

In terms of dosage, all studies provided specific information about the amount of omega-3 or omega-6 fatty acids used, with the highest dose being 1.5 g/day (16) and the lowest dose being 200 mg/day of DHA (25).

When examining the difference between omega-3 supplementation alone and a combination of omega-3 and omega-6, the analysis of four studies that exclusively utilized omega-3 supplementation yielded outcomes that were varied and inconclusive. Amminger et al. (16) and Doaei et al. (21) reported significant improvements in ASD symptoms with the use of omega-3 alone, while Voigt et al. (25) and Bent et al. (17) found no significant improvement.

Studies using a combination of omega-3 and omega-6 also showed mixed results. Boone et al. (19) and Keim et al. (24) observed significant improvements in caregiver-reported behavior, sleep, and ASD symptoms. Additionally, Sheppard et al. (22) reported an increase in gesture use in the treatment group. However, Mazahery et al. (23), who used a combination of omega-3 and Vitamin D, found no significant improvement.

## Discussion

Our study shows that irrespective of the criteria used to assess diverse outcomes–whether it pertains to the effectiveness of omega-3 alone versus a combination of omega-3 and omega-6, varying dosages, or differing durations of supplementation–the enhancement of behavioral symptoms in children with autism remains inconclusive. This ambiguity could potentially stem from a multitude of factors, including the specific dosage administered, the duration of the treatment, the particular symptoms targeted for improvement, and the inherent individual differences among the children. Furthermore, it's important to acknowledge that some studies incorporated additional elements like vitamin D or omega-9 in their combinations, introducing the possibility of these elements also exerting an influence on the observed outcomes (23).

The observed differences in the effects of omega-3-6 supplementation on ASD symptoms and behaviors could be attributed to several factors.

First, variations in the characteristics of the study populations, such as age range, severity of ASD symptoms, and comorbidities, can contribute to the heterogeneity of the findings. The response to omega-3 supplementation may vary among individuals with different profiles, leading to contrasting outcomes across studies.

Second, differences in the dosage and duration of omega-3-6-9 supplementation may influence the results. Studies have utilized varying dosages of omega-3 fatty acids and administered them for different lengths of time, which can impact the efficacy and magnitude of any observed effects. The optimal dosage and duration of supplementation are yet to be determined.

Additionally, the choice of outcome measures and assessment tools used in the studies can contribute to the discrepant findings. There is a lack of standardized measures for assessing ASD symptoms and behaviors, which can lead to variability in the interpretation of results. The use of diverse assessment tools across studies may affect the ability to compare and combine the findings. These measures encompassed a range of variables, such as autism symptom severity, maladaptive behaviors, social interaction, communication, psychopathology, behavior and sleep in toddlers, plasma and erythrocyte FA profiles, and inflammation markers. The variability in outcome measures adds complexity to the interpretation of the results and underscores the need for standardized assessments in future studies.

Moreover, variations in the composition and quality of the omega-3 supplements used in the studies may also contribute to the differences in outcomes. The types and ratios of omega-3 fatty acids, as well as the purity and formulation of the supplements, can vary between studies, potentially influencing their effectiveness.

## Gaps, clinical implications, and future research

The current landscape of research on the use of essential fatty acids (EFAs) in ASD presents an intricate web of possibilities and challenges. The significant strides made in understanding the impact of omega-3 and omega-6 on ASD symptoms have opened the door to broader investigations. However, the complexity of ASD, combined with the multifaceted nature of EFAs, introduces a series of gaps that need to be explored further.

A prominent gap is observed in the investigation of the role of EFAs in other neurodevelopmental disorders including attention deficit disorder and ADHD. The heterogeneity of ASDs and their frequent co-occurrence with other conditions introduces a layer of complexity that necessitates a broader perspective.

Moreover, comparisons between EFAs' impact on ASDs' core symptoms and other complementary and alternative medications, such as oxytocin, secretin, elimination diets, or other biomedical treatments, offer an avenue for future exploration. Such a comparative analysis could unravel nuanced understandings of EFAs' unique contributions and synergistic effects.

The current literature also falls short of studies examining EFAs in combination with early intervention programs as opposed to stand-alone interventions. The interplay between EFAs and other therapeutic measures could lead to more personalized and effective strategies, opening new horizons in clinical practice.

Further in-depth analysis of the potential role of EFAs in psychiatric comorbidities in ASDs vs. ASDs without comorbidities could shed light on specific applications and tailored interventions. This could be particularly illuminating, given the established literature on EFAs' role in depressive disorders.

Another vital aspect is the assessment of the isolated effect of EFAs from conventional medications and therapy, especially regarding hyperactivity and restlessness in children with ASD and ADHD. Such isolation could provide clearer insights into the specific benefits of EFAs.

A broader economic evaluation concerning the cost-effectiveness of EFA use also emerges as a significant gap. Such an assessment could guide policy and practice, aligning therapeutic choices with economic realities.

Finally, an exploration of the short-term vs. long-term side effects of EFA use, as well as the necessary monitoring, needs to be integrated into the research framework. The holistic understanding of EFAs' impact necessitates a balance between benefits and potential risks.

## Limitations

It is important to note that the evidence from these studies is diverse and not definitive. The sample sizes also varied, with some studies having small sample sizes. Additionally, the publication dates ranged from 2007 to 2022, indicating a span of research over several years.

Additionally, methodological differences, such as study design, sample size, blinding, and control groups, can impact the robustness and reliability of the findings. Studies with larger sample sizes, well-controlled designs, and appropriate blinding are generally considered to provide more reliable results.

Our review also uncovers additional limitations that warrant mention. An understanding of the role EFAs play in improving outcome measures is complicated by a lack of isolation from conventional medications and therapy. This intermingling calls for separate investigations that unravel EFAs' unique contributions. The benefits of EFA in children with ASD and ADHD warrant separate attention, particularly in improving hyperactivity and restlessness. The potential overlap and distinctiveness between ASD and ADHD in response to EFAs require nuanced exploration. There is a need for more studies to assess the cost-effectiveness of EFA use and evaluate the short-term vs. long-term side effects and the monitoring required.

## Conclusion

While both omega-3 alone and a combination of omega-3 and omega-6 have shown potential benefits in some studies, more research is needed to definitively understand their relative effectiveness in treating behavioral symptoms associated with autism. Considering many additional factors, it is crucial to interpret the findings of the studies on omega-3-6-9 supplementation in ASD with caution and acknowledge the complexities and nuances involved in understanding the effects of supplementation on ASD symptoms and behaviors. More randomized controlled clinical trials with longer follow-up periods that address these factors and utilize rigorous methodologies are needed to provide more definitive conclusions.

## Author contributions

RP conceived the idea for this article and identifying the need to explore a specific topic within the field of study. RP, SN, AS, KZ, DZ, and LD collaborated in retrieving relevant articles and conducting extensive research. SN took the lead in developing the methodology and devising a robust framework for data analysis and interpretation. The collective efforts of all authors contributed to the comprehensive exploration of the subject matter and the generation of insightful findings. All authors contributed to the article and approved the submitted version.
